# Comments on Sigmoid Volvulus

**DOI:** 10.4314/ejhs.v34i5.12

**Published:** 2024-09

**Authors:** Sabri Selcuk Atamanalp

**Affiliations:** 1 Department of General Surgery, Faculty of Medicine, Ataturk University, Erzurum, Türkiye

Dear Editor,

I have reviewed the paper by Awedew et al. (1) on the treatment of gangrenous sigmoid volvulus (SV). Although SV is not widespread globally, it is prevalent in our region of eastern Anatolia (2,3). Over 57.5 years (from June 1966 to the present), my team and I have treated 1,076 SV patients, representing the largest single-center series worldwide (3). Based on this extensive experience, I offer the following comments on the surgical management of SV.

Firstly, Awedew et al. reported lower mortality and higher morbidity rates for patients with gangrenous SV who were treated with resection and primary anastomosis compared to those treated with resection and Hartmann's stoma. Their evaluation extensively referenced our 952-case dataset published in 2013 (4). In our updated dataset of 285 gangrenous SV patients out of a total of 1,076 cases, we treated 171 patients with a stoma (157 Hartmann's and 14 Mikulicz's) and 114 with primary anastomosis (48 primary anastomosis and 66 primary anastomosis with operative colonic lavage). The mortality rates for the stoma and primary anastomosis groups were 26.3% (45 patients) and 17.5% (20 patients), respectively. Similarly, the morbidity rates were 39.8% (68 patients) and 33.3% (38 patients), respectively. Our current mortality data align with the findings of Awedew et al. (1), but our morbidity data differ. This discrepancy might be attributed to the limited number of studies on morbidity, comprising only five in total.

Secondly, the authors proposed a new treatment protocol incorporating ASA, ECOG scores, the presence of organ dysfunction, shock, and the feasibility of a tension-free anastomosis. They compared their protocol with the Atamanalp classification and treatment algorithm I presented in 2020 (5). While a large number of parameters may complicate practical application, I appreciate the authors' new protocol. However, considering that life expectancy varies by region and has increased over time, a life expectancy-based assessment may be more appropriate than a fixed age limit of 60 years. For example, with a life expectancy of 75 years in our region, the 60-year threshold might be too low. I recommend this consideration for the authors' future updates.

I commend the authors for their work and look forward to their response to my comments.

**Author Response** (Awedew AF)

I want to express my sincere appreciation for your valuable insights and recommendations. I have come to recognize the profound expertise of Dr. Atamanalp and his colleagues in the management of sigmoid volvulus (SV). Their contributions have been instrumental in shaping practical recommendations for addressing SV cases. It is evident that SV is prevalent in regions often referred to as the “Volvulus belt,” encompassing countries like Turkey, the Middle East, and various parts of Africa, particularly in Eastern Africa, including countries like Ethiopia and Kenya. In Ethiopia, SV stands as the most common cause of bowel obstruction. Despite its prevalence, there remains a significant gap in the development of guidelines and a comprehensive analysis akin to the practices seen among surgeons in Turkey. Acknowledging this disparity, we have endeavored to fill this void by synthesizing existing studies to generate a robust evidence base for the management of SV, aiming to elevate the standards of care and decision-making in regions affected by this condition.

Based on our assessment, the lower rate of morbidity observed in our review can be attributed to the limited number of studies that actually reported on morbidity rates. We anticipate that future updates of our review will incorporate fresh research findings, allowing us to conduct a more thorough analysis.

In terms of the age limit discussion, we sincerely appreciate your perspective and the insightful comments you have provided. Our decision to set the age limit at 60 years stemmed from our firm belief that the life expectancy in Low- and Middle-Income Countries (LMICs) generally hovers in the 60s range. Given that sigmoid volvulus (SV) is more prevalent in the so-called “volvulus belt” countries, where life expectancy typically remains within the 60s, we felt this cutoff was justified.

Your feedback is truly valuable, and I want to assure you that we will take your comments into serious consideration. Rest assured that the age limit criteria, particularly in relation to life expectancy, will be revisited and potentially revised in the forthcoming update of our protocol.

Your input is integral to the refinement and enhancement of our research approach.
